# Drug Reaction With Eosinophilia and Systemic Symptoms (DRESS) Syndrome in a Polymedicated Patient With Osteomyelitis: A Diagnostic Challenge

**DOI:** 10.7759/cureus.116009

**Published:** 2026-09-09

**Authors:** Molly E Milano, Darius Ghahary, Arie Rennert

**Affiliations:** 1 Family Medicine, Sidney Kimmel Medical College, Philadelphia, USA; 2 Family Medicine, Morristown Medical Center, Morristown, USA

**Keywords:** drug reaction, drug reaction with eosinophilia and systemic symptoms (dress) syndrome, lamotrigine toxicity, osteomyelitis, polypharmacy

## Abstract

Drug reaction with eosinophilia and systemic symptoms (DRESS) is a rare but serious drug reaction that can be life-threatening. This report reviews a case of a male in his 30s with a history of extensive burn injuries, osteomyelitis, and bipolar disorder who was admitted to the hospital for left foot osteomyelitis after failing home intravenous (IV) piperacillin-tazobactam treatment. On hospital day 4, the patient began to develop a progressive macular rash. Laboratory results were significant for worsening transaminitis and elevated eosinophil levels, concerning for DRESS. Active inpatient medications included aripiprazole, lamotrigine, vancomycin, and meropenem. Lamotrigine and all antibiotics were discontinued in the setting of suspected DRESS. Treatment was initiated with IV methylprednisolone 175 mg daily, with a taper over eight to 12 weeks, and an infusion of N-acetylcysteine for liver injury, with eventual improvement of liver enzymes to baseline and resolution of eosinophilia. This case underscores the importance of prompt recognition of DRESS in patients with polypharmacy and complex infectious disease management.

## Introduction

Drug reaction with eosinophilia and systemic symptoms (DRESS) is a rare and potentially life-threatening drug reaction that occurs as a result of a severe delayed T-cell-mediated reaction [1]. The typical presentation of DRESS includes a diffuse maculopapular rash, recurring fevers, organ involvement such as the liver and kidneys, and hematological findings of eosinophilia and atypical lymphocytosis [1]. Reactivation of herpes viruses is often a complication of such a reaction [2]. Diagnostic criteria for DRESS syndrome include those proposed by the Registry of Severe Cutaneous Adverse Reactions (RegiSCAR), which include clinical and laboratory findings that lend themselves to a scoring system with possible outcomes of “a negative case,” “a possible case,” “a probable case,” and “a definitive case” of DRESS [3]. The reported incidence of DRESS varies based on individual patient factors as well as the suspected causative drug. The estimated risk is anywhere from one in 1,000 to one in 10,000 [4]. Symptoms of DRESS usually occur anywhere from two to six weeks after drug exposure [5]. Drugs most commonly associated with DRESS include antiepileptics like phenytoin, lamotrigine, carbamazepine, and phenobarbital; sulfonamides; sulfones like dapsone; antituberculosis agents; nonsteroidal anti-inflammatory drugs (NSAIDs) like piroxicam, ibuprofen, and diclofenac; beta-lactam antibiotics; vancomycin; allopurinol; minocycline; and antiretrovirals [6-12]. Mortality rates documented in the literature range from 3.8% to 10%, with the main cause of mortality due to DRESS occurring secondary to fulminant hepatitis and liver necrosis [6,13]. The varying presentations and delayed onset of symptoms of DRESS can make its prompt diagnosis challenging, especially in the disease’s early phases [14-16]. The capability of DRESS to cause severe organ damage and even death makes prompt diagnosis paramount. Treatment of DRESS includes immediate discontinuation of the offending drug, as well as administration of systemic corticosteroids as first-line treatment [17]. The following report will describe a case of DRESS syndrome in a male in his 30s, discuss potential causal drugs, and detail the importance of early recognition of this rare drug reaction.

## Case presentation

A male in his thirties with a history of extensive burn injuries as a teenager (complicated by peripheral neuropathy, recurrent osteomyelitis, and prior skin grafts, left toe osteotomy, and right great toe amputation), pericarditis, bipolar disorder, and major depressive disorder presented with fevers (39.7°C), chills, and persistent left foot pain. He had been discharged two weeks prior on intravenous (IV) piperacillin-tazobactam via a peripherally inserted central catheter (PICC) line for foot osteomyelitis. Home medications included aripiprazole, duloxetine, lamotrigine, and piperacillin-tazobactam.

On arrival, he was tachycardic (106 beats per minute (bpm)) with leukopenia (white blood cell count (WBC) of 2.30/nL) and a lactate level of 2.2 mmol/L, meeting sepsis criteria. Laboratory values on presentation are included in Table 1. Examination of the left foot revealed great toe erythema and a plantar ulcer. Computed tomography (CT) with contrast demonstrated an ill-defined hypodensity beneath the ulcer, consistent with phlegmonous changes. He was admitted for sepsis secondary to osteomyelitis, despite outpatient IV antibiotics.

**Table 1:** Patient laboratory studies on presentation AST: aspartate aminotransferase; ALT: alanine aminotransferase; WBC: white blood cell count; RBC: red blood cell count; ESR: erythrocyte sedimentation rate; CRP: C-reactive protein

Laboratory study	Value	Reference range
AST	151 U/L	8-48 U/L
ALT	129 U/L	7-56 U/L
WBC	2.30/nL	4.5-11/nL
RBC	4.05/pL	4.20-5.70/pL
Platelets	110/nL	150-450/nL
ESR	12 mm/hr	<15 mm/hr
CRP	86.4 mg/dL	<0.3 mg/dL

Vancomycin 1500 mg was initiated in the emergency department (ED), and infectious disease broadened coverage to IV meropenem 1 gram every eight hours (q8h) and IV vancomycin 1500 mg q8h. On hospital day 2, podiatry performed partial amputation of the left great toe and left second digit with bone biopsy. Intraoperative wound cultures grew coagulase-negative Staphylococcus and Klebsiella. Despite source control (amputation and subsequent PICC line removal) and broad-spectrum antibiotics, fevers persisted with consistently negative blood cultures. A transthoracic echocardiogram (TTE) was obtained to evaluate for endocarditis and was initially read as concerning for a possible vegetation. However, cardiology recommended deferring transesophageal echocardiography (TEE) given the negative blood cultures and the patient's current broad-spectrum antibiotic regimen.

On hospital day 4, a palpable, violaceous rash appeared on the bilateral lower extremities in the distribution of the sequential compression devices (SCDs), which subsequently progressed to the chest, abdomen, and back (Figures 1, 2).

**Figure 1:**
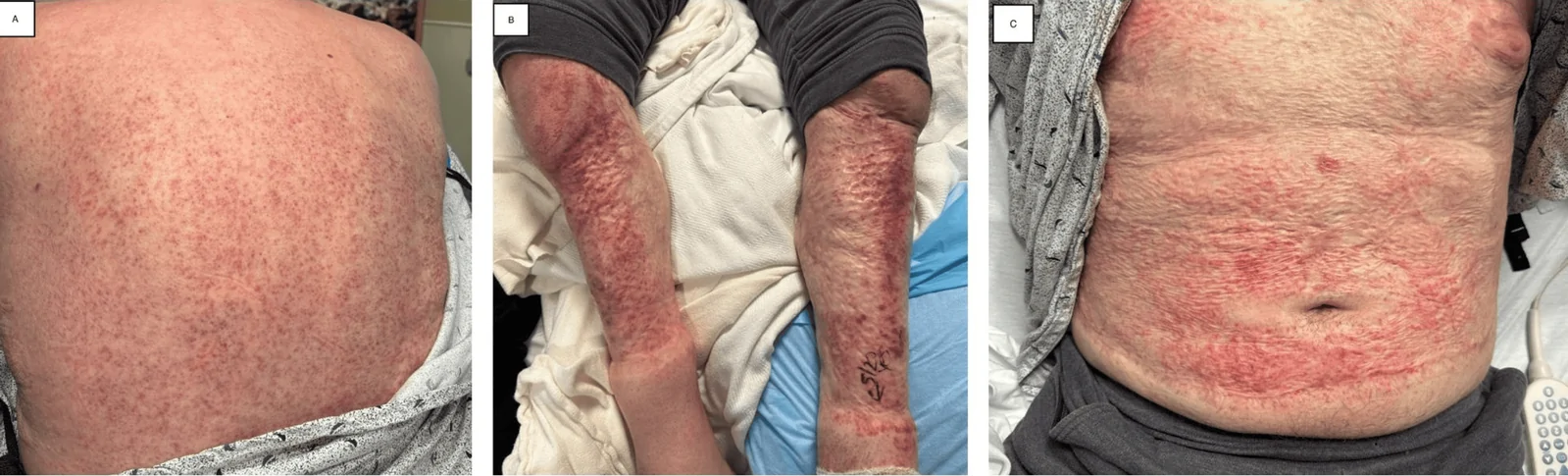
Diffuse rash emerging on day 4 of hospitalization Diffuse erythematous maculopapular rash with areas of purpuric discoloration. (A) Rash involving the back. (B) Rash involving both lower extremities. (C) Rash involving the chest and abdomen

**Figure 2:**
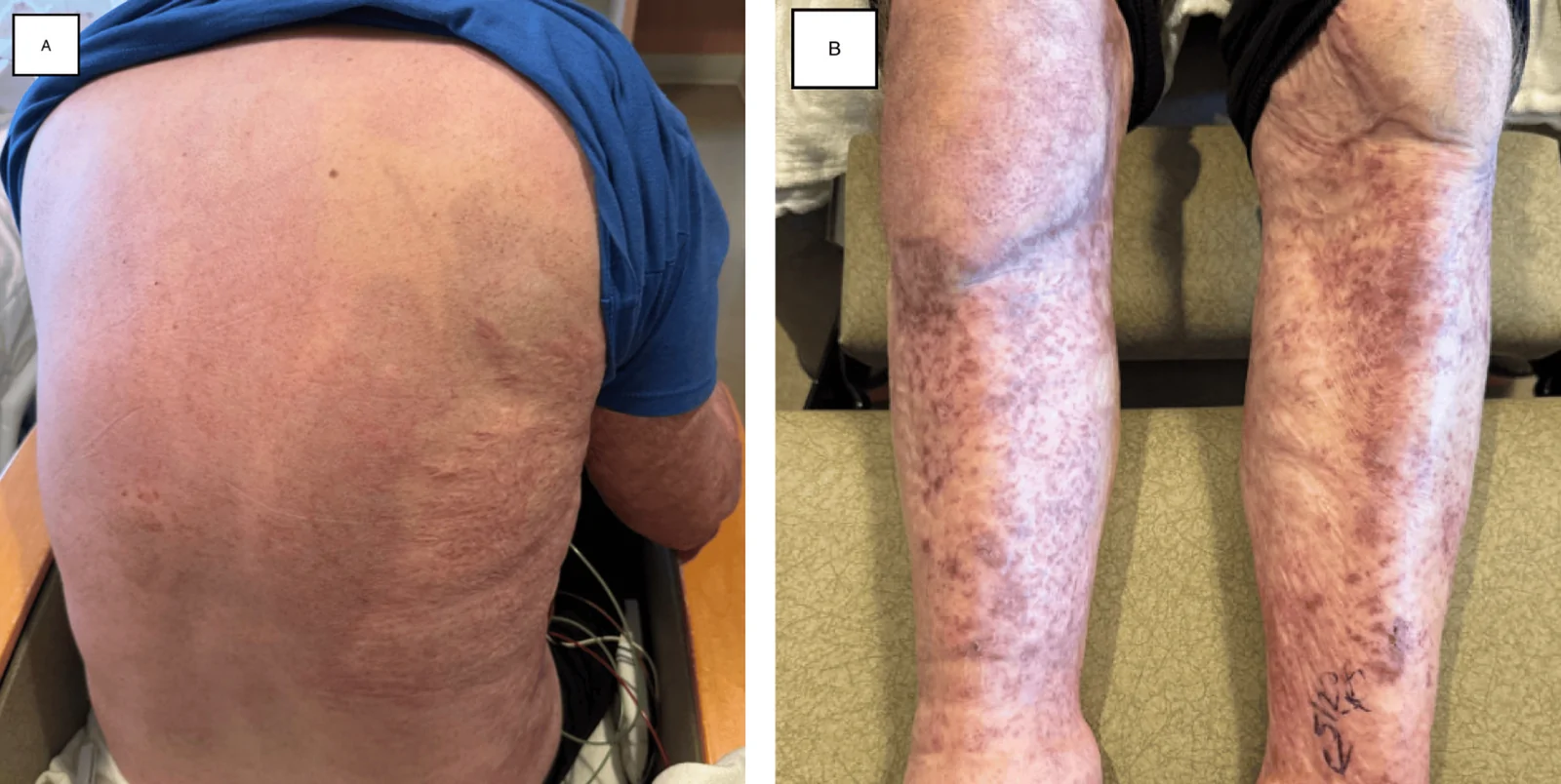
Improvement in rash after 24 hours of treatment with IV methylprednisolone (A) Improving rash on the back. (B) Improving rash on bilateral lower extremities. IV: intravenous

Meropenem was switched to cefepime with initial suspicion of a drug-related rash. By hospital days 5-7, the patient developed eosinophilia (eosinophil percentage 14.8%; ref 0.0-8.0%) and severe transaminitis (aspartate aminotransferase (AST) peak 1,423 U/L, alanine aminotransferase (ALT) peak 2,077 U/L) (Table 2).

**Table 2:** Patient laboratory studies on hospital day 5 AST: aspartate aminotransferase; ALT: alanine aminotransferase

Laboratory study	Value	Reference range
AST	1,423 U/L	8-48 U/L
ALT	2,077 U/L	7-56 U/L
Eosinophilia percent	14.8%	0.0-8.0%

Workup for transaminitis included serologies for Ehrlichia, Anaplasma, Epstein-Barr virus (EBV), cytomegalovirus (CMV), herpes simplex virus (HSV) 1/2, viral hepatitis, and human immunodeficiency virus (HIV), all of which were negative. Right upper quadrant ultrasound with Doppler was ordered to assess for possible septic emboli in the setting of potential endocarditis but showed no evidence of septic emboli or portal vein thrombosis. Renal function remained normal without proteinuria. Given the negative infectious and vascular workup, acute liver injury was initially attributed to piperacillin-tazobactam exposure.

Cardiology re-reviewed the TTE and determined that the previously reported vegetation was very unlikely to represent a vegetation, given both its appearance and the absence of valvular dysfunction. In consultation with infectious disease, the patient met no major and only one minor Duke criterion (fever), making infective endocarditis overwhelmingly unlikely. Surgical pathology from the amputation was pending at the time of clinical decision-making, although podiatry's intraoperative assessment confirmed resection to clear margins.

The initial differential for our patient's skin changes included petechiae from SCD compression in the setting of thrombocytopenia (platelet count, 110/μL), but this was excluded when the rash spread beyond the compression sites. Stevens-Johnson syndrome was considered given recent lamotrigine initiation and widespread distribution, but the absence of prodromal flu-like symptoms, blistering, or epidermal detachment made this diagnosis less likely. DRESS syndrome was ultimately diagnosed based on the constellation of widespread maculopapular rash, eosinophilia, intermittent fevers, and severe transaminitis in the setting of a patient receiving multiple high-risk medications (lamotrigine, piperacillin-tazobactam, vancomycin, and meropenem). The patient's RegiSCAR score of 4 made the likelihood of a DRESS diagnosis probable.

Given the patient’s complex medication regimen and exposure to multiple potential culprit medications, the Naranjo Adverse Drug Reaction Probability Scale was applied separately to each suspected agent to assess the relative likelihood of drug-induced causality (Table 3) [18]. All potential offending medications were discontinued. Lamotrigine and aripiprazole were stopped immediately; duloxetine was tapered (30 mg daily×3 days, then 20 mg daily×3 days, then discontinued) per psychiatry. All IV antibiotics were discontinued after reevaluation of the negative blood cultures, unlikely endocarditis, and intraoperative confirmation of clear surgical margins, all of which made infection as the source of worsening transaminitis unlikely.

**Table 3:** Naranjo adverse drug reaction probability scale assessment of potential causative medications The Naranjo adverse drug reaction probability scale was applied separately to each potential causative medication to assess the likelihood of drug-related causality. Scores were interpreted as follows: ≥9, definite; 5-8, probable; 1-4, possible; and ≤0, doubtful.

Suspected medication	Criteria applied	Naranjo score	Interpretation
Piperacillin-tazobactam	Temporal relationship, improvement upon drug withdrawal, alternative causes, objective evidence, and prior reports	3	Possible
Vancomycin	Temporal relationship, improvement upon drug withdrawal, alternative causes, objective evidence, and prior reports	3	Possible
Meropenem	Temporal relationship, improvement upon drug withdrawal, alternative causes, objective evidence, and prior reports	3	Possible
Cefepime	Initiated after rash onset, alternative causes, and objective evidence	0	Doubtful
Lamotrigine	Temporal relationship, improvement upon drug withdrawal, alternative causes, objective evidence, and prior reports	3	Possible
Aripiprazole	Temporal relationship, improvement upon drug withdrawal, alternative causes, and objective evidence	2	Possible
Duloxetine	Temporal relationship, improvement upon drug withdrawal, alternative causes, and objective evidence	2	Possible

The patient was started on IV methylprednisolone 175 mg daily, with improvement in the rash noted within 24 hours (Figure 2).

Once liver enzymes began to improve, steroids were transitioned to oral prednisone 0.5-1 mg/kg/day with a planned eight- to 12-week taper. Gastroenterology recommended a 48-hour IV N-acetylcysteine infusion for hepatoprotection. Despite the severe transaminitis and thrombocytopenia, the international normalized ratio (INR) remained normal, and the patient had no altered mental status, making acute liver failure unlikely.

Following discontinuation of all antibiotics and initiation of corticosteroids, the patient's clinical status improved rapidly. He was discharged home once liver function tests were definitively downtrending, with outpatient follow-up planned for steroid taper monitoring and serial liver function testing.

Informed consent was obtained from the patient for publication of this case and accompanying images.

## Discussion

DRESS is a condition resulting from an adverse drug reaction that typically begins within the first two months after initiation of an offending drug and is characterized by fever, malaise, and facial edema or dermatitis [19]. The onset most commonly occurs two- to six-weeks after the drug is initiated; however, it can occur at any point following drug administration, and the most common drugs include antiepileptic drugs and sulfonamides [19]. The rash is symmetric and typically starts as a morbilliform eruption on the face, upper trunk, and upper extremities and will continue to worsen if the offending drug is not discontinued, with additional symptoms potentially involving the mucous membranes, lymph nodes, or elevated temperatures (drug fever) [19]. The proposed diagnostic criteria involve 1) a cutaneous drug eruption, 2) hematologic abnormalities (eosinophilia ≥1500/μL or atypical lymphocytes), and 3) systemic involvement, and diagnosis is confirmed if three criteria are present [19]. However, while these diagnostic criteria are found in the literature, it may be more accurate to use a more comprehensive scoring system. A more comprehensive scoring system to help diagnose DRESS is the RegiSCAR for DRESS, first proposed in 2007. This scoring system takes into account several objective measures and produces a numerical scale to address the likelihood of a DRESS diagnosis. The present patient met several of the aforementioned criteria for DRESS syndrome, and thus the diagnosis of DRESS was made. The rash and hepatic involvement that may be seen in DRESS may persist for weeks after the drug is discontinued, and management is typically with systemic glucocorticoids such as prednisone 0.5 mg/kg per day [20].

DRESS has been reported to be T-cell-mediated and associated with relapse two to four weeks following acute symptoms, often coinciding with reactivation of prevalent chronic persistent human herpesviruses such as human herpesvirus 6, EBV, and CMV. DRESS has also been reported to have a 10% mortality rate, often related to unrecognized myocarditis and CMV complications [1]. Viral reactivation is often addressed in reviews regarding this condition and is not only common but also associated with poor outcomes and increased mortality [20]. The possibility of HSV reactivation was something the primary team was concerned about during the patient’s hospital admission, as our patient began to develop oral lesions suggestive of HSV; however, testing was negative. The concern that the patient may have been experiencing viral reactivation increased the urgency of establishing the proper treatment plan, making the present case especially challenging.

The rate of recurrence of DRESS is not frequently discussed in the literature. Although recurrence is not completely understood, it is very important to be cognizant of the possibility of recurrence due to its increasing potential for morbidity and mortality. Because of the lack of consensus regarding the recurrence of DRESS syndrome in the literature, the clinical team advised our patient not to take lamotrigine again due to concerns that it may be associated with DRESS recurrence. Furthermore, this case was complicated by the degree of our patient's polypharmacy, especially in the realm of antibiotic therapy. Our patient was started on multiple different antibiotics, so future antibiotic treatment will require careful monitoring for symptoms concerning for DRESS, especially in a patient who has known chronic osteomyelitis, which often requires antibiotic therapy. Interestingly, a literature review by Sharma et al. identified 42 articles involving a total of 60 patients and found that relapse was most often attributed to the introduction of a new medication, with the survival rate of those experiencing recurrence being around 71% [21]. The results of this study illuminate the complicated nature of our patient's case and the future medical management of his multiple comorbid conditions. Mood stabilizers and antibiotics are common causal agents of DRESS syndrome, making medical management of our patient more complicated than that of others. Our patient is a young man whose mental health conditions require mood-stabilizing medications and whose history of extensive burns and subsequent skin grafting renders him more susceptible to wound infections and osteomyelitis than the general population. His medical comorbidities necessitate management with multiple drug classes frequently implicated in DRESS syndrome, underscoring the need for careful multidisciplinary follow-up going forward.

DRESS management is guided by severity. DRESS may be considered mild, moderate, or severe based on clinical criteria, and management ranges from high- to super-high-potency topical corticosteroids to systemic steroids, which require a taper to help avoid relapse [17]. Other immunosuppressants may need to be used if the condition is refractory to systemic steroids. Ultimately, the management of DRESS is well studied, but there are varying degrees of clinical presentation, and the condition itself requires a carefully thought-out approach based on various patient factors and possible organ involvement.

The present case is unique in that our patient was simultaneously on a number of potential causal drugs implicated in cases of DRESS syndrome. Moreover, this case was particularly challenging because broad-spectrum antibiotics remained clinically indicated given concerns for bacteremia and possible endocarditis, yet the very medications required to treat his presumed infectious process may have been the offending agents driving his DRESS syndrome. This diagnostic and therapeutic tension was further compounded by persistently negative blood cultures and worsening laboratory values despite continued antibiotic therapy, ultimately forcing the team to weigh the risks of discontinuing empiric coverage against the harm of prolonged exposure to potential causative drugs. The specific causal drug was difficult to identify, but cessation of all potential causative drugs, including lamotrigine, vancomycin, meropenem, and piperacillin-tazobactam, which are well-documented in the literature, became a necessity. The patient was ultimately treated appropriately and achieved both subjective and objective improvement.

## Conclusions

This case underscores the importance of interdisciplinary collaboration in the healthcare setting, as a number of specialists were involved in the management of our patient in order to achieve the best possible clinical outcome. Long-term treatment plans for our patient become challenging because patients who experience DRESS due to a particular medication or antibiotic are generally not recommended to use that same drug again in the future due to the risk of recurrence, potential worsening of symptoms upon re-exposure, and increasing difficulty in managing recurrent DRESS. The exact offending medication was difficult to identify in our patient’s case, making future considerations of using lamotrigine, vancomycin, piperacillin-tazobactam, and meropenem riskier than for most other patients who have experienced DRESS syndrome after being placed on one offending medication. Prompt recognition of DRESS and subsequent discontinuation of potentially offending drugs are the most effective ways to improve morbidity and mortality secondary to DRESS syndrome. Polypharmacy in the inpatient hospital setting is a frequent occurrence and may make drug-related reactions difficult to manage.
